# **Cloning and characterization of thermophilic endoglucanase and its application in the transformation of ginsenosides**

**DOI:** 10.1186/s13568-022-01473-z

**Published:** 2022-10-28

**Authors:** Fei Zheng, Huanxi Zhao, Nan Wang, Peng Zhong, Kailu Zhou, Shanshan Yu

**Affiliations:** Jilin Ginseng Academy, Changchun University of Chinese Medicine, 130117 Changchun, China

**Keywords:** Biotransformation, Endoglucanase, Ginsenoside, Glycoside hydrolase family, *Fervidobaterium pennivorans* DSM9078

## Abstract

A novel endoglucanase (BcelFp) was identified from *Fervidobaterium pennivorans* DSM9078 which had biotransformation activity for protopanaxadiol (PPD)-type ginsenosides. Sequence analysis of BcelFp revealed that it could be classified into glycoside hydrolase family 5 (GH5). The gene encoding a 323-amino acid protein was cloned and expressed in *Escherichia coli.* The recombinant enzyme was purified, and its molecular weight was approximate 37 kDa. The recombinant BcelFp exhibited an optimal activity at 95 ^o^C and pH 5.5 and showed high thermostability. The endoglucanase had high selectivity for cleaving the outer glucose moiety at the C3 carbon of ginsenoside Rb1, Rb2, Rc and Rd, which produced stronger pharmacologically active gypenoside XVII (GypXVII), Compound O (CO), Compound Mc1 (CMc1) and F2, respectively. The K_m_ values for Rb1, Rb2, Rc and Rd were 3.66 ± 0.04 µM, 4.02 ± 0.12 µM, 5.95 ± 0.03 µM, 0.67 ± 0.006 µM, respectively. The k_cat_/K_m_ value of BcelFp for ginsenoside Rd was 27.91 mM^-1^s^-1^, which was much higher than that of the previously enzymes. This study was the first report of the highly efficient and selective transformation of GypXVII, CO, CMc1 and F2 from Rb1, Rb2, Rc and Rd by a GH5-family thermophilic endoglucanase.

**Supplementary Information:**

The online version contains supplementary material available at 10.1186/s13568-022-01473-z.

## Introduction

Ginseng, the root of the *Panax* ginseng C. A. Mey., has been widely used as a kind of valuable Chinese-traditional medicine in the Eastern Asian for thousands of years. Ginsenosides are the principal components responsible for the diverse and significant effects of ginseng (Shi et al. 2019). The concentration of gypenoside XVII (GypXVII), compound O (CO), compound Mc1 (CMc1), and F2 are quite low in Ginseng. Whereas these four ginsenosides exhibit various pharmaceutical activities. GypXVII exerts strong cardio-protective (Yu et al. 2021), anti-apoptotic and autophagic activities (Sun et al. 2021) and protects against reperfusion and spinal cord injury (Luo et al. 2021). Ginsenoside F2 possesses anti-obesity (Siraj et al. 2015) and anti-cancer activity (Shin et al. 2012). There are very few researches about pharmaceutical activities of CO and CMc1 due to the production difficulty. Therefore, the industrial preparation of these ginsenosides will lay an important foundation for their pharmacological research and application development.

**Fig. 1:**
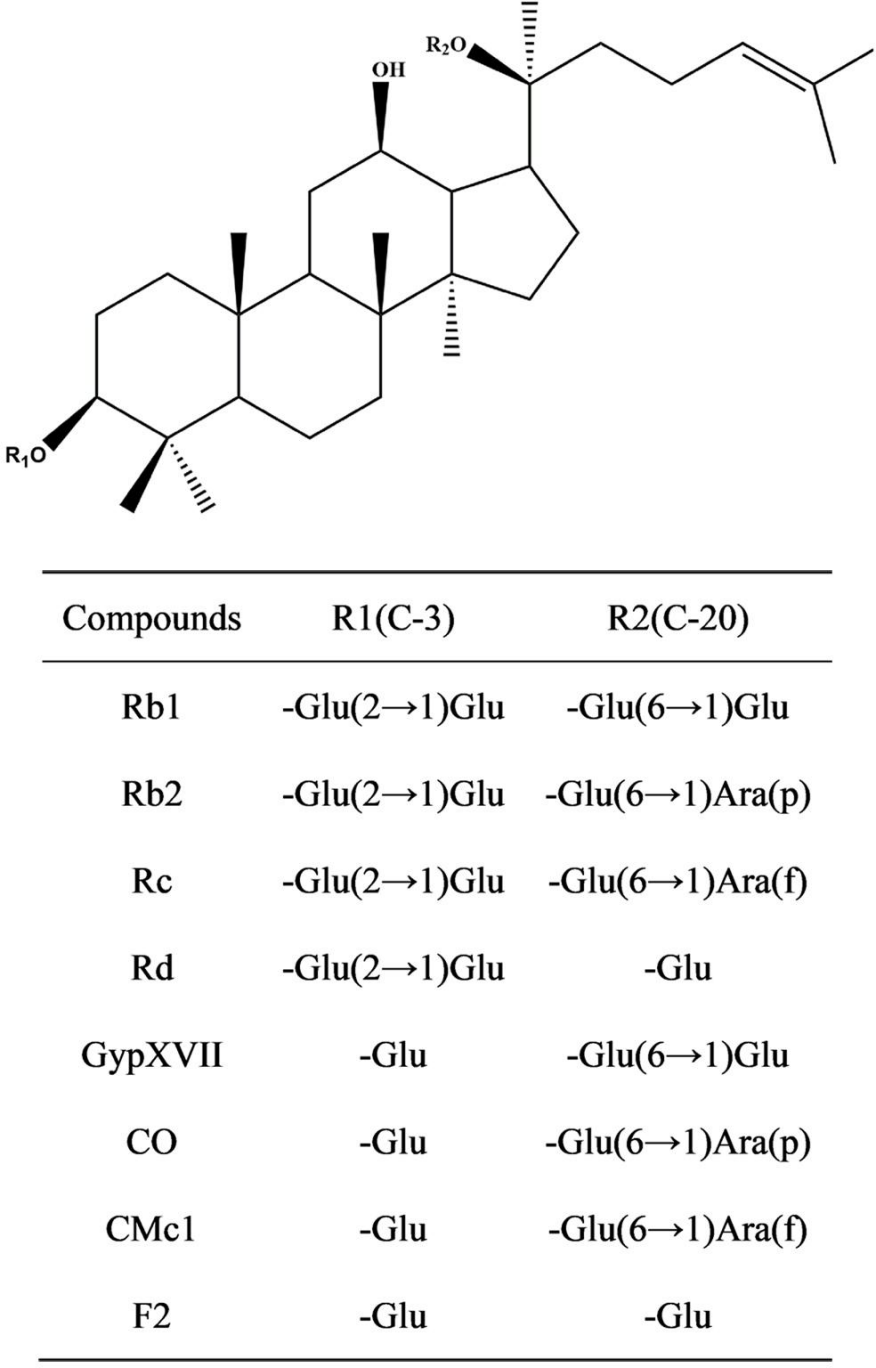
Chemical structures of PPD-type ginsenosides. *Glu* β-D-glucopyranosyl, *Arap* α-L-arabinopyranosyl, *Araf* α-L-arabinofuranosyl

Ginsenosides comprise a nonsugar constituent of dammarane skeleton and a sugar constituent including 1–4 molecule glycosides such as glucose, arabinopyranose, arabinofuranose, xylose, and rhamnose (Fig. 1) (Park et al. 2010). Based on the principle of structural similarity of ginsenosides, GypXVII, CO, CMc1, and F2 can be generated by hydrolysis of the outer glucose linked to the C3 position of ginsenoside Rb1, Rb2, Rc, and Rd, respectively. Ginsenoside Rb1, Rb2, Rc and Rd can be easily extracted from ginseng roots. They possess the same sugar moieties linked to C3 position in aglycon PPD. Both the C3 inner and outer sugars are glucose. The structural differences of these four ginsenosides lie in the sugar moieties linked to C20 position. The C20 inner sugar is glucose and the C20 outer sugar is glucose, arabinopyranose, xylose or arabinofuranose (Park et al. 2010). Therefore, the aim of our work is to transform glycosylated ginsenosides (Rb1, Rb2, Rc and Rd) through hydrolysis of the sugar moieties to deglycosylated ginsenosides (GypXVII, CO, CMc1, and F2).

Deglycosylated ginsenosides have been shown to have greater pharmacological activity than glycosylated ginsenosides because of their smaller size, better permeability across cell membranes, and higher bioavailability (Ku 2016). In our previous work, we demonstrated that deglycosylated ginsenosides (Rd, GypXVII, and PPT) had significantly greater anti-inflammatory activity than their glycosylated precursors (Rb1, Re and Rg1) (Yu et al. 2017). Hence, transformation of ginsenosides have attracted wide attention.

Various transformation approaches such as physiochemical methods including heating, acid treatment and alkali treatment, and bioconversion methods using microorganisms and enzymes have been attempted (Park et al. 2010). Among these methods, the enzymatic conversion is the most promising method because of its high substrate specificity and stability, low levels of by-products, and high production yields. To minimize processing time and production cost, recombinant enzymes obtained from *Escherichia coli* (*E. coli)* strains are commonly applied for the ginsenoside conversion. For instance, the recombinant β-glucosidase from *Bifidobacterium breve* ATCC 15,700 was identified to produce compound K(CK) from ginsenoside Rd (Zhang et al. 2019). Higher productivity was achieved when recombinant thermophilic enzymes were used to transform ginsenosides. The thermophilic β-glycosidase from *Sulfolobus acidocaldarius* converted 1 mg/ml reagent grade Rb1, Rb2, and Rc to 0.53 mg/ml compound K, 0.56 mg/ml compound Y, and 0.70 mg/ml compound Mc, respectively, corresponding to mole conversion yields of 94, 80, and 100% (mol/mol), respectively (Noh et al. 2009).

Until now, many glycosidases have been explored to hydrolyze the sugar moieties linked to the C3, C6, and C20 positions in glycosylated ginsenosides. These ginsenoside-transforming glycosidases include β-glucosidases, arabinopyranosidases, arabinofuranosidases, xylosidases, rhamnosidases and β-galactosidases, belonging to glycoside hydrolase (GH) family 1, 2, 3, 39, 42, 51, and 78 (Shin and Oh 2016). For instance, The GH1 β-glucosidase from *Sphingopyxis alaskensis* specifically hydrolyzed the outer glucose at the C3 position in PPD-type ginsenosides (Shin and Oh 2014). Thermophilic glycosidases from GH family 1, 3, 39 and 42 were also identified to transform PPD- or PPT- type ginsenosides (Noh and Oh. 2009; Pei et al. 2015; Xie et al. 2015; Shin et al. 2013). Considering the complex and diverse structures of ginsenosides, the number of ginsenoside-transforming glycosidases is still limited. And the biotransformation activity, specificity, and thermostability of most enzymes still do not meet the industrial demands. It is therefore meaningful to explore novel glycosidases with good thermostability, high catalytic efficiency and specificity.

In this paper, a novel thermophilic endoglucanase from *Fervidobacterium. pennivorans* DSM9078 was cloned, purified, and characterized. It showed high specificity that hydrolyzed only the outer glucose at the C3 position in PPD-type ginsenosides, and biotransformed the glycosylated ginsenoside Rb1, Rb2, Rc, and Rd to the deglycosylated ginsenoside GypXVII, CO, CMc1, and F2, respectively. This study was the first report of the highly efficient and selective transformation of PPD-type ginsenosides by a GH5-family thermophilic endoglucanase.

## Materials and methods

### Materials

*Escherichia coli* strains were incubated at 37 °C in Luria-Bertani (LB) medium (10 g/L tryptone, 5 g/L yeast extract, and 10 g/L NaCl) with 50 mg/L kanamycin. Reference standard of ginsenoside Rb1, Rb2, Rc, Rd, GypXVII and F2 were purchased from Shanghai Yuanye Biological Technology Co. Ltd. (Shanghai, China).

### Sequnce analysis of BcelFp

The theoretical molecular weight (Mw) and isoelectric point (pI) of BcelFp were estimated on the ExPasy server (http://web.expasy.org/computepi/). (Gasteiger et al. 2005) Homologs of BcelFp (Genbank No. AFG35892.1) were searched with the BLASTp program on NCBI (https://blast.ncbi.nlm.nih.gov/Blast.cgi). The glycoside hydrolases in GH5 family were chosen at the CAZy web (http://www.cazy.org/fam/GH5.html), and the sequence information of those proteins were collected by means of the CAZy web page links. Multiple alignments of BcelFp and three characterized glycosidases from GH5 family were performed using the ClustalX program (Larkin et al. 2007). The phylogenetic tree of BcelFp and the enzymes chosen from GH5 family was constructed using the neighbor-joining method (Saitou and Nei 1987) with default parameters and 1000 bootstrap in the MEGA4 Program (Felsenstein 1985).

### Molecular cloning, expression and purification of BcelFp

Genomic DNA from *F. pennivorans* DSM9078 was extracted using a genomic DNA extraction kit (TIANGEN, Beijing, China) and used as a template of the gene cloning. The endoglucanase gene, *bcelfp*, from *F. pennivorans* DSM9078 was amplifed via polymerase chain reaction (PCR). The primers were designed based on genomic sequence (Genbank No. WP_245530410.1): upstream (5’-CAGCAGGGATCCATGGATCAGTCAGTTGCT-3’) and downstream (5’- CAGCAGCTCGAGTTATTCTTTGCTTTCTCCAA-3’) with *Bam*HI and *Xho*I restriction sites (underlined), respectively. The PCR amplified DNA fragment with a His-tag at N terminal was purified and inserted into the pET28a vector digested with *Bam*HI and *Xho*I. The recombinant pET28a-*bcelfp* was transformed into *E. coli* BL21-CodonPlus(DE3)-RIL electrocompetent cells. Cells were grown at 37 °C in LB medium supplemented with 50 µg^.^ml^− 1^ kanamycin until the OD600 reached 0.6–0.8; protein expression was induced by the addition of 1 mM isopropyl *β*-D-thiogalactopyranoside (IPTG) and the culture was grown for 12 h at 16 °C.

The recombinant strains were collected by centrifugation at 6,000×g for 20 min and resuspended in a lysis buffer (50 mM Tris-HCl, pH 7.1). The cells were then sonicated and centrifuged at 14,000×g for 30 min at 4 ^o^C to remove the debris. The supernatants containing the target proteins were loaded onto a Ni-NTA affinity chromatography column (GE Healthcare) and purified using a 20–100 mM imidazole gradient. The purified enzyme was dialyzed against 50 mM phosphate-citrate buffer (pH 6.0) and concentrated to 1.0 mg/mL. The protein homogeneity was confirmed by 10% sodium dodecyl sulfate polyacrylamide gel electrophoresis (SDS-PAGE).

### Endoglucanase activity assay of BcelFp

The hydrolytic activity of the endoglucanase BcelFp was assayed according to the Ghose (Ghose 1987) and DNS (Miller 1959) methods. Protein concentrations were determined by using the Bradford Protein Assay Kit (Sangon Biotech, Shanghai, China). The reaction mixture (2 ml) composed of 1% carboxymethyl cellulose sodium salt (CMC) (w/v) and 1 mg/ml endoglucanase BcelFp in 50 mM phosphate-citrate buffer (pH 6.0). After incubation at 95 ^o^C for 5 min, 3,5-Dinitrosalicylic acid (DNS) was added to terminate the action, and the mixture was boiled in 100 ^o^C water for 5 min. The absorption of the reaction mixture was measured at 540 nm. One unit (IU) of enzyme activity was defined as the amount of enzyme that released 1 µmol reducing sugars per min.

### Enzyme characterization of BcelFp

Using CMC as the substrate, the effects of temperature and pH on enzyme activity were investigated by assaying the endoglucanase activity in a 2 ml reaction mixture according to the method described previously. The pH optima of BcelFp was tested over the pH range of 4.0–8.0 in 50 mM phosphate-citrate buffer. The temperature optima of BcelFp was measured between 30 and 100 ^o^C (5 ^o^C intervals) at the optimum pH. Thermal stability of BcelFp was studied by incubating about 1.5 mg/mL purified enzyme solutions at 85 ^o^C, 90 ^o^C or 95 ^o^C for various lengths of time in 50 mM phosphate-citrate buffer (pH 6.0). The residual activity on CMC was determined at 95 ^o^C in 50 mM phosphate-citrate buffer (pH 6.0). pH stability of BcelFp was tested by incubating about 1.5 mg/mL purified enzyme solutions in pH 4.0, pH 5.0, pH 6.0 and pH 7.0 for various lengths of time at 85 ^o^C. The residual activity on CMC was determined at 95 ^o^C in 50 mM phosphate-citrate buffer (pH 6.0).

The effects of metal ions (NH^4+^, Na^+^, K^+^, Co^2+^, Ca^2+^, Zn^2+^, Ba^2+^, Mn^2+^, and Fe^3+^), and EDTA on enzyme activity were investigated. The enzyme activity with no addition for the control was set as 100%. After incubation the enzyme with various metal ions (5 mM and 10 mM), and EDTA (5 mM and 10 mM) at room temperature for 30 min, the residual activity was measured according to the method described previously using CMC as substrate at pH 6.0 and 95 ^o^C in a 2 ml reaction mixture.

### Differential scanning calorimetry (DSC)

Thermal inactivation of BcelFp was analyzed by DSC on a VP-Capillary differential scanning calorimeter (MicroCal, LLC, GE Healthcare) over a temperature range from 35 to 120 ^o^C. The purified enzyme was dialyzed against 50 mM PBS buffer (pH 6.0) and concentrated to 1.5 mg/mL. The corresponding buffer was used as a reference. The equilibrated enzyme was scanned at a rate of 2.0 K/min. The scans were analyzed after subtraction of an instrument baseline recorded with buffer in both cells using the software package Origin provided by the manufacturer.

### Substrate specificity of BcelFp

The substrate specificity of BcelFp was investigated by using the following polysaccharide substrates: CMC (carboxymethyl cellulose sodium salt, medium viscosity; Fluka), RAC (regenerated amorphous cellulose) (Zhang et al. 2006), Avicel (PH-101; Fluka), β-glucan from barley (Sigma), laminarin (Sigma), soluble starch (Sigma), and pustulan (Sigma). The hydrolytic activities of the BcelFp were measured after 5 or 30 min of incubation at 95 ^o^C in 50 mM phosphate-citrate buffer (pH 6.0) in the presence of 1% (w/v) polysaccharide substrates (Ghose 1987; Miller 1959). One unit (IU) of enzyme activity was defined as the amount of enzyme that released 1 µmol reducing sugars per min.

The *p*-nitrophenyl β-d-glucopyranoside (*p*NPG) was used to test the β-glucosidase activity of BcelFp. The hydrolytic reaction was performed at 95 ^o^C in 50 mM phosphate–citrate buffer (pH 6.0) containing 1 mM *p*NPG and 1 mg/ml enzyme. The activity was determined by measuring the increase in absorbance at 405 nm due to the release of *p*-nitrophenol. One unit (IU) of activity was defined as the amount of enzyme liberating 1 µmol of *p*-nitrophenol per min.

The substrate specificity of BcelFp towards different PPD- and PPT-type ginsenosides were also studied. In reaction mixture (2 ml), enzyme solution (2 mg/mL) was reacted with equal volume of ginsenoside solution (1 mg/mL) in 50 mM phosphate–citrate buffer (pH 6.0) at 95 °C for 5–30 min. The mixture was then extracted with 2 ml n-butanol for 2 min using a vortex. After centrifugation at 13,000×g for 5 min, the upper organic phase as n-butanol fraction was transferred into clean tube and evaporated to dryness in the centrifugal evaporator. The residue was then reconstituted by methanol for analysis. Ginsenosides were assayed by the High Performance Liquid Chromatography (HPLC) system (Agilent Technologies, Santa Clara, CA, USA) coupled with a Syncronis C_18_ chromatographic column (5 cm×3.0 mm, 2.7 μm, Supelco. USA). The column oven temperature was maintained at 35 °C, and the mobile phases A and B were water with 0.1% formic acid and acetonitrile, respectively. The gradient elution program was determined as follows: 0-2.5 min, 19% (B); 2.5-5 min, 19–30% (B); 5–11 min, 30–33% (B); 11–20 min, 33–45% (B); 20–25 min, 45–65% (B). The injection volume was 5 µL, and the flow rate was 0.4 mL/min. One unit (IU) of activity was defined as the amount of enzyme catalyzing the conversion of 1 µmol of ginsenoside substrate per min.

### Biotransformation of PPD-type ginsenosides by BcelFp

The biotransformation ability of recombinant BcelFp towards PPD-type ginsenosides was studied. Ginsenoside Rb1, Rb2, Rc, and Rd were used as substrates. In reaction mixture (2 ml), enzyme solution (2 mg/mL) was reacted with equal volume of ginsenoside solution (1 mg/mL) in 50 mM phosphate–citrate buffer (pH 6.0) at 95 °C. The reaction solution without enzyme served as blank control. Samples were obtained at regular intervals and analyzed via HPLC.

Kinetic studies were performed by using Rb1, Rb2, Rc and Rd at concentrations from 0.5 to 10.0 mM in 50 mM phosphate–citrate buffer (pH 6.0) at 95 °C. One unit (IU) of activity was defined as the amount of protein required to convert 1 µmol of substrate per min. All assays were performed in triplicate. The parameters K_m_ and V_max_ were determined using the enzyme kinetics program described by Cleland (Cleland 1979).

### HPLC-Q-TOF-MS/MS analysis

Chromatographic separation was performed on an Agilent 1200 series HPLC system (Agilent Technologies, Santa Clara, CA, USA) coupled with a Syncronis C_18_ chromatographic column (5 cm×3.0 mm, 2.7 μm, Supelco. USA). The column oven temperature was maintained at 35 °C, and the mobile phases A and B were water with 0.1% formic acid and acetonitrile, respectively. The gradient elution program was determined as follows: 0-2.5 min, 19% (B); 2.5-5 min, 19–30% (B); 5–11 min, 30–33% (B); 11–20 min, 33–45% (B); 20–25 min, 45–65% (B). The injection volume was 5 µL, and the flow rate was 0.4 mL/min.

Mass spectrometric detection was carried out on an Agilent 6520 Q-TOF-MS/MS (Agilent Technologies, Santa Clara, CA, USA) equipped with electrospray ionization source operated. The scan range for MS acquisition was from 100 to 2200 *m/z* in negative ionization mode. The parameters of ion source were set as follows: drying gas, N_2_; flow rate, 4.0 mL/min; drying gas temperature, 350 ^o^C; nebulizer, 30 psig; capillary voltage, 3500 V; fragmentor, 350 V; cone voltages, 65 V. Data analysis was performed by Mass Hunter Qualitative (MHQ) software, version B.03.01 (Agilent Technologies, Santa Clara, CA, USA).

### ^**13**^** C NMR analysis**

The biotransformed products were isolated using a Waters prep-HPLC system (Waters, USA) with a Waters Sunfire Prep C_18_ OBD^™^ column (19 mm×50 mm, 5 μm). Isocratic elution was performed at room temperature using a mixture of H_2_O/ACN (56:44, v/v) for product 1, H_2_O/ACN (48:52, v/v) for product 2, H_2_O/ACN (54:46, v/v) for product 3 and H_2_O/ACN (54:46, v/v) for product 4, respectively. The flow rate was 15 mL/min, and the UV detection was at 203 nm. The injection volume was set at 200 µL. The purity of the products were evaluated by HPLC in an Agilent 1200 series HPLC system (Agilent Technologies, USA). The separation conditions were the same as that used in the HPLC analysis. The ^13^ C NMR spectra of the purified products were recorded on a Bruker AVIII 600 MHz spectrometer at an operating frequency of 151 MHz (Bruker BioSpin, Germany).

## Results

### **Sequence analysis of BcelFp from*F. pennivorans*DSM9078**

**Fig. 2:**
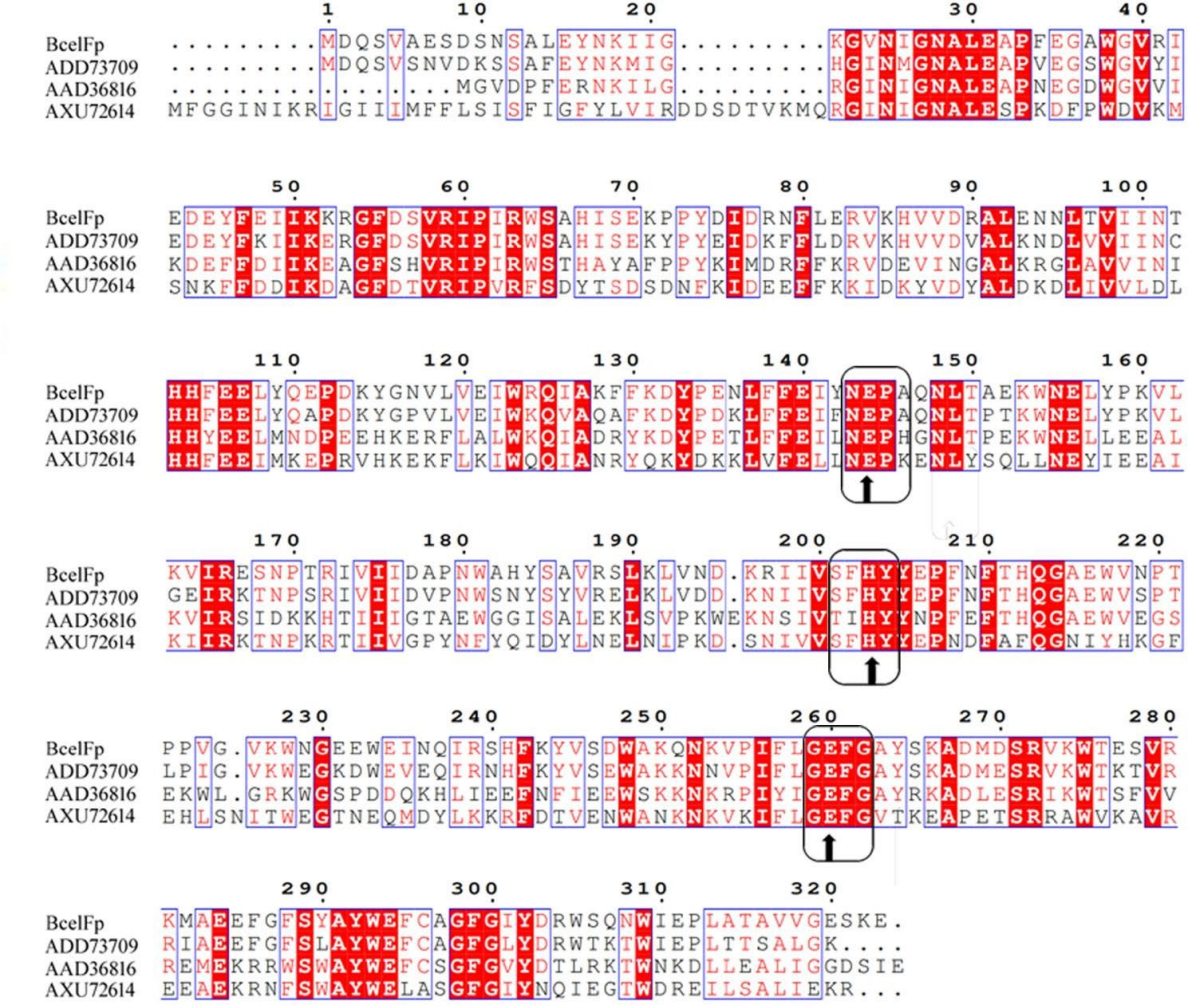
Multiple amino acid sequence alignment of BcelFp with several characterized glycoside hydrolases from GH5. The accession numbers of the aligned sequences are for the following organisms: ADD73709, endoglucanase FnCel5A from *Fervidobacterium nodosum* Rt17-B1; AAD36816, endoglucanase from *Thermotoga maritima* MSB8; AXU72614. endoglucanase from *Clostridioides difficile*. The accession numbers were indicated to the left of the amino acid sequences. Identical residues are indicated by a red background. Symbols: ↑ amino acids forming a catalytic residues

The endoglucanase gene *BcelFp* consisted of 969 bp encoding 323 amino acids with a theoretical molecular mass of 37.89 kDa and a theoretical pI value of 5.43. The amino acid sequence of BcelFp (Genbank No. AFG35892.1) exhibited the highest similarity with GH 5 proteins from *Fervidobacterium islandicum* (85.7% identity, Genbank No. WP_052107242.1 ) and *Fervidobacterium changbaicum* (85.7% identity, Genbank No. WP_090223359.1). These proteins have not yet been characterized. The nearest characterized glycoside hydrolase (76% identity, GenBank No. WP_011994708.1) in the CAZy database was cellulase FnCel5A from *Fervidobacterium nodosum*. The BcelFp and FnCel5A were both from thermophilic bacteriums belonging to genus *Fervidobacterium.* FnCel5A was the first cellulase of the genus *Fervidobacterium* that had been cloned and expressed (Wang et al. 2010). The alignment of BcelFp with several characterized glycoside hydrolases from GH5 indicated that these proteins shared some conserved peptide motifs, namely NEP (residues 143–145), HYY (residues 203–205) and GEFG (residues 259–262) (Fig. 2). The Glu144 and Glu260 residues were typical catalytic residues of the GH5 enzymes, which confirmed that BcelFp belonged to GH5 family (Aspeborg et al. 2012).

**Fig. 3:**
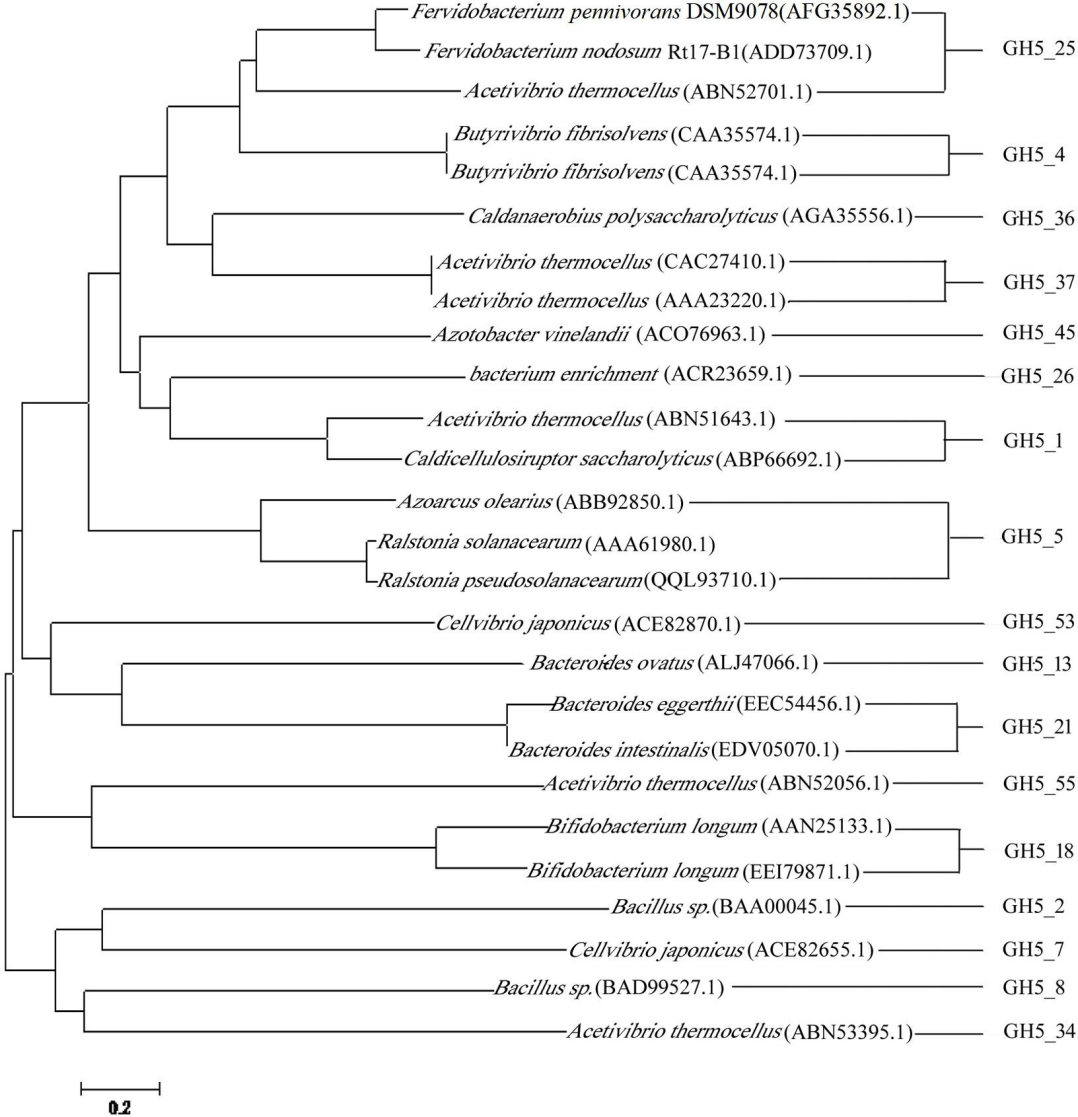
Phylogenetic analysis of BcelFp, and other characterized glycoside hydrolases from GH5. The units at the bottom of the tree indicate numbers of substitution events

In order to gain a better understanding of the evolutionary position of BcelFp in glycoside hydrolase family 5, we constructed the phylogenetic tree using the neighbor-joining method in the MEGA4 program with bootstrap values based on 1,000 replications. The resulting consensus tree is presented in Fig. 3. BcelFp from *Fervidobacterium pennivorans* DSM9078 clustered within subfamily 25 and formed a separate, well-supported clade with cellulase from *Acetivibrio thermocellus* and endoglucanase (FnCel5A) from *Fervidobacterium nodosum* Rt17-B1.

### Expression, purification and characterization of the endoglucanase BcelFp

The putative endoglucanase gene from *F. pennivorans* DSM9078 was cloned and expressed in *Escherichia coli* under the control of the IPTG-inducible promoter T7. After being induced under 16 ^o^C for 12 h with 1 mM IPTG, the recombinant enzyme was solubly overexpressed in *E.coli* cells. The recombinant BcelFp was purified by His-trap affinity chromatography with a final purification of 2.3-fold and a specific activity of 297 U/mg for CMC. The expressed enzyme was determined as a single band by SDS-PAGE, with a molecular mass of approximately 38 kDa (See Additional file 1: Fig. S1), which was almost consistent with the molecular weight of 37,892 Da calculated from 323 amino acids.

**Fig. 4:**
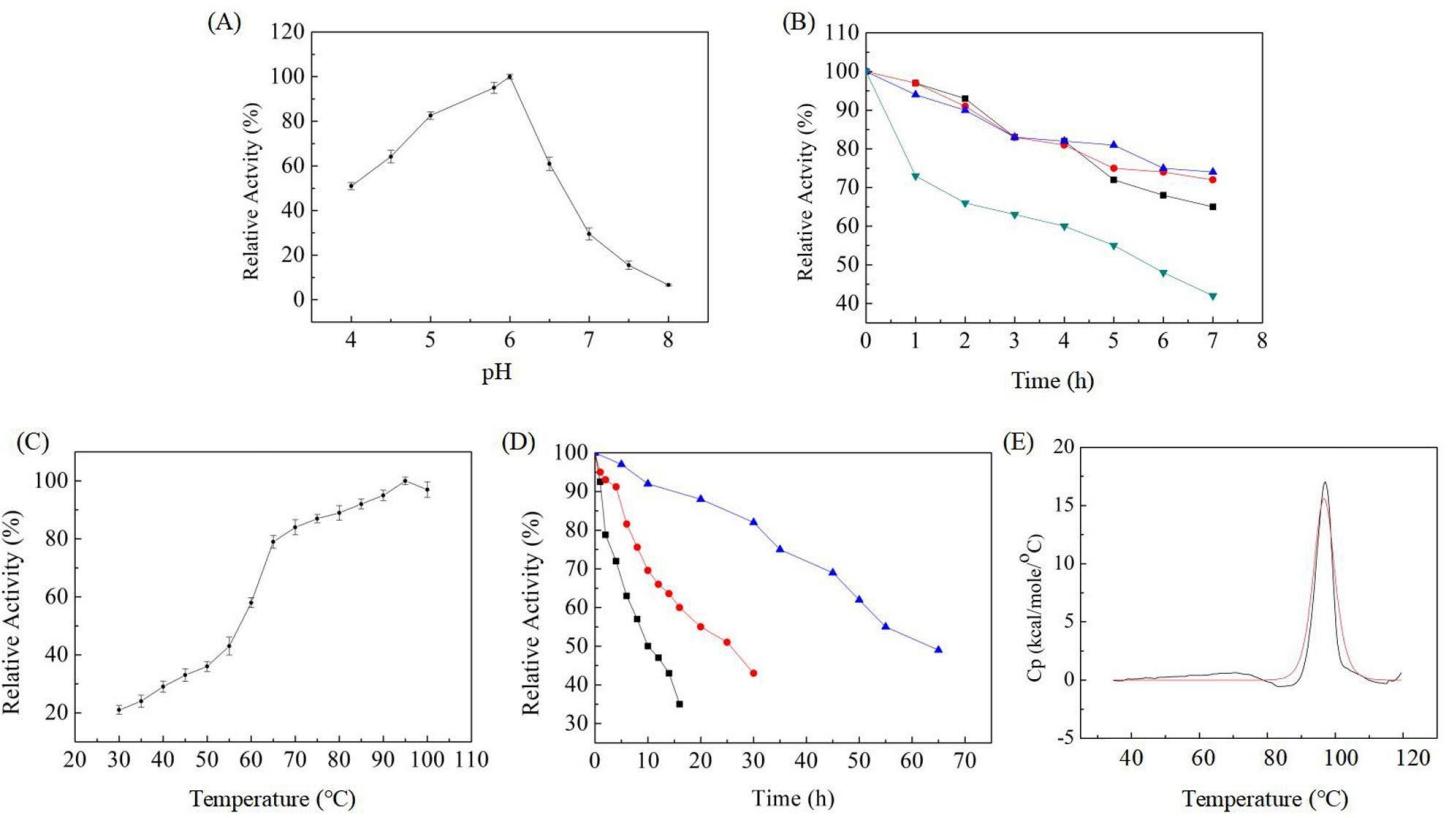
(A) Effect of pH on enzyme activity. (B) Effect of pH on enzyme stability. The activities were determined by assays with CMC as substrate following incubation of the enzyme at pH 4 (■), pH5(●), pH 6 (▶) and pH 7 (◀) for the indicated times. (C) Effect of temperature on enzyme activity. (D) Effect of temperature on enzyme stability. The activities were determined by assays with CMC as substrate following incubation of the enzyme at 85 °C (▲), 90 °C (●), and 95 °C (■) for the indicated times. (E) Differential Scanning Calorimetry (DSC) analysis of BcelFp. The enzyme was concentrated to 1.5 mg/ml in 50 mM PBS buffer (pH 6.0). The equilibrated enzyme was scanned from 35 to 120 ^o^C at a rate of 2.0 K/min. The enzyme scan was corrected using a buffer–buffer baseline

The optimum pH and temperature of the purified BcelFp were determined using CMC as the substrate. The maximum activity was observed at pH 6.0, and the activity was higher than 50% of the maximum activity in the range of pH 4.0 to 6.5. While from pH 6.5, the enzyme activity decreased swiftly, representing that the enzyme was active over narrow acid pH range (Fig. 4 A). After being incubated at various pHs (pH 4.0, pH 5.0, pH 6.0 and pH 7.0) for 1 h, more than 90% of enzyme activity remained at acid pHs, while 70% of enzyme activity remained at pH 7.0, indicating that BcelFp was more stable at acid pHs (Fig. 4B). The optimal temperature for BcelFp activity was 95 ^o^C, while it also displayed high activity between 60 and 100 ^o^C (Fig. 4 C). Hence, the BcelFp was thermophilic enzyme.

**Fig. 5:**
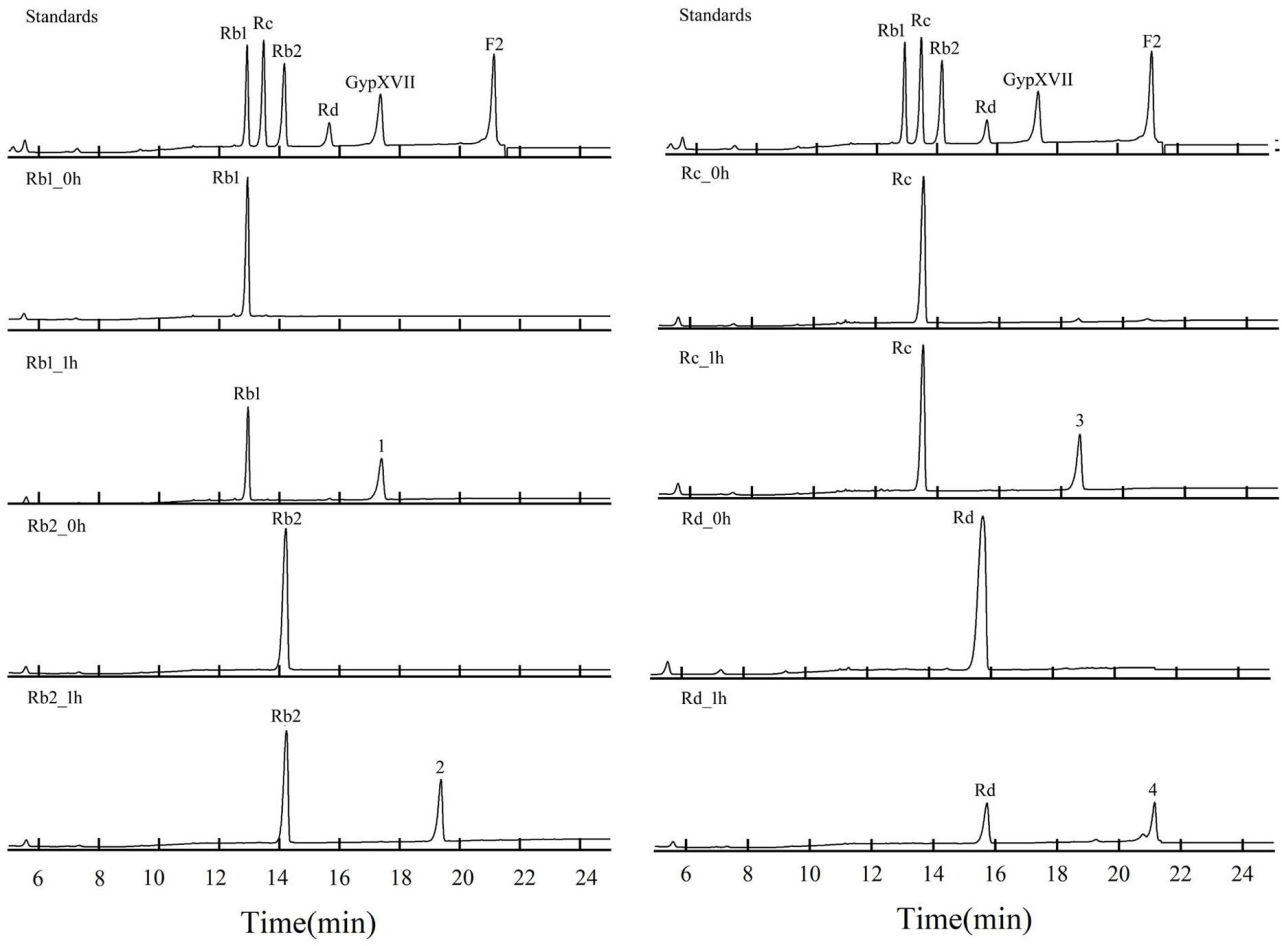
HPLC analysis of ginsenoside Rb1, Rb2, Rc and Rd during biotransformation process by using BcelFp. Ginsenoside standards were indicated on the peaks. Numbers were used to indicate the product peaks

**Fig. 6:**
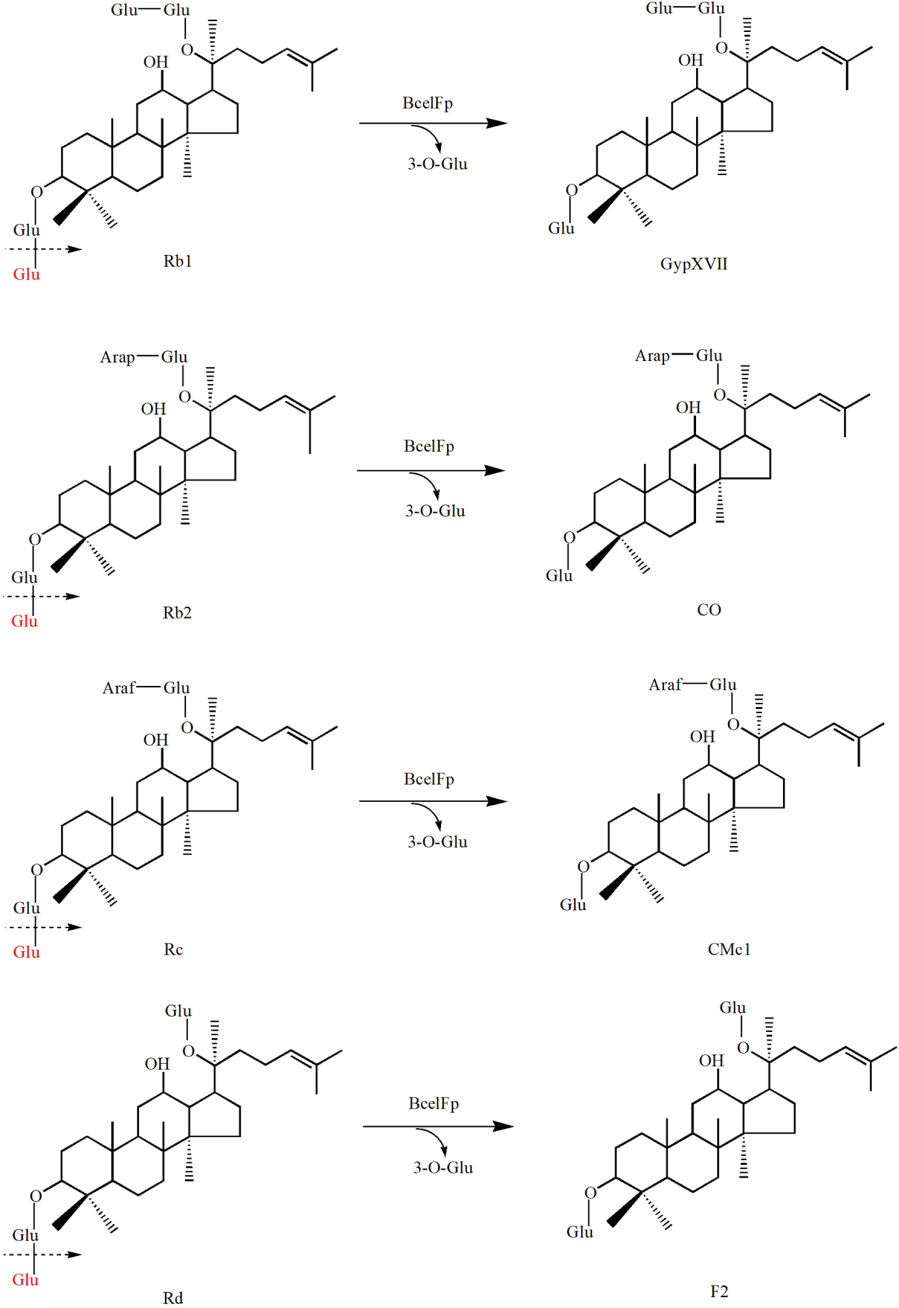
Biotransformation pathways of ginsenoside Rb1, Rb2, Rc and Rd by using BcelFp.

The effect of temperature on enzyme stability was investigated using CMC as the substrate. As seen in Fig. 4D, the enzyme was incubated for various lengths of time at 85, 90 and 95 ^o^C at pH 6.0 and then the residual activities were measured. The enzyme was very stable at high temperature, and its half-lives at 85, 90 and 95 ^o^C were 60, 25, and 10 h, respectively. When DSC analysis was performed to determine the thermal transition mid-point (Tm) of BcelFp, it was 96 ^o^C (Fig. 4E).

The effects of metal ions, and EDTA on BcelFp activity were also investigated (Table 1). Na^+^ and Mn^2+^ were able to significantly increase enzyme activity under the high concentration of 10 mM. Moreover, Fe^3+^, Co^2+^, Zn^2+^ were able to inhibit enzyme activity under concentrations of 5 mM and 10 mM. No significant effect was observed in the presence of Ca^2+^, NH^4+^, Ba^2+^, and K^+^ under the high concentration of 10 mM. The chelating agent EDTA did not inhibit the activity, which indicated that BcelFp was not metalloprotein.

**Table 1:** Effects of metal ions and EDTA on the enzyme activity of BcelFp

Additives	Relative Activity (%)	
Control	100	100
Metal ions	5 mM	10 mM
NH_4_Cl	102.8 ± 0.8	108.7 ± 0.4
NaCl	108.0 ± 2.5	117.0 ± 0.6
BaCl_2_	78.2 ± 2.2	93.9 ± 1.0
KCl	92.3 ± 0.3	103.8 ± 0.5
MnCl_2_	95.7 ± 1.7	143.8 ± 0.6
CaCl_2_	103.0 ± 0.5	108.0 ± 0.6
ZnCl_2_	91.0 ± 0.4	33.9 ± 0.4
CoCl_2_	85.1 ± 0.2	56.0 ± 0.5
FeCl_3_	32.2 ± 0.5	29.8 ± 0.8
Inhibitors	5 mM	10 mM
EDTA	87.8 ± 1.5	95.7 ± 0.9

### Substrate specificity of the endoglucanase BcelFp

The substrate specificity of BcelFp was investigated using substrates including CMC, barley β-glucan, RAC, Avicel, Laminarin, soluble starch, pustulan, and *p*NPG. CMC is typical substrate for determining endoglucanase activity, while Avicel is used for measuring exoglucanase activity (Percival Zhang et al. 2006). The *p*NPG is a synthetic compound which serves as an optimal substrate for β-glucosidase. As seen in Table 2, BcelFp displayed high activity towards CMC, but undetectable activity on Avicel and *p*NPG, therefore it was an endoglucanase. Moreover, BcelFp was able to hydrolyze RAC, structure of which was similar to CMC. In addition, selectivity of BcelFp on β-1,6-, β-1,3-, ɑ-1,4-, ɑ-1,6- linkages in glycosides were also tested. Barley β-glucan is composed of a mixed-linked β-1,3/1,4-glucans. Laminarin contains primarily β-1,3 and a portion of β-1,6-glycosidic bonds. Soluble starch consists of ɑ-1,4/1,6-glucans. Pustulan is a linear β-1,6-linked glucans. BcelFp exhibited highest activity on barley β-glucan, while no activity on laminarin, soluble starch, and pustulan, which further indicating that BcelFp can randomly hydrolyze the β-1,4-glucopyranosyl linkage, but cannot act on β-1,6-, β-1,3-, ɑ-1,4-, ɑ-1,6- linkages in glycosides.

**Table 2:** Enzyme specificity for BcelFp on various substrates

Substrate	Relative Activity(%)
CMC	100^a^
Barley β-glucan	486
RAC	21
Avicel	ND^b^
Laminarin	ND
Soluble starch	ND
pustulan	ND
*p*NPG	ND

a The activity against CMC was assumed to be 100%, and corresponded to a specific activity of 297 U/mg

b Not detected, specific activity is not detected by the analytical methods used in this study

The substrate specificity of BcelFp on PPD- and PPT-type ginsenosides was also measured. As seen in Table 3, the sugar moieties including glucose, arabinopyranose, and arabinofuranose linked to C3 and C20 position in PPD-type ginsenosides. And the sugar moieties including glucose, xylose, and rhamnose linked to C6 and C20 position of PPT-type ginsenosides. The relative activity of BcelFp for the PPD-type ginsenosides was in the order Rd > Rb1 > Rb2 > Rc. However, BcelFp showed undetectable activity on GypXVII, CO, CMc1, F2, and Rg3. In addition, for PPT-type ginsenosides as substrates, the enzyme exhibited no activity on R1, Re, Rg1, Rg2 and Rh1, indicating that BcelFp didn’t hydrolyze sugar moieties linked to the C6 and C20 position of PPT-type ginsenosides.

**Table 3:** Substrate specificity of BcelFp on PPD- and PPT-type ginsenosides

Type	Substrate^a^	C3	C6	C20	Relative Activity (%) (mean % ± SD)^b^
PPD-	Rb1	Glu(1→2)Glu-		Glu(1→6)Glu-	68.18 ± 1.51
	Rb2	Glu(1→2)Glu-		Arap(1→6)Glu-	27.27 ± 3.28
	Rc	Glu(1→2)Glu-		Araf(1→6)Glu-	25.18 ± 0.73
	Rd	Glu(1→2)Glu-		Glu-	100 ± 1.27
	Rg3	Glu(1→2)Glu-		H-	ND^c^
	GypXVII	Glu-		Glu(1→6)Glu-	ND
	CO	Glu-		Arap(1→6)Glu-	ND
	CMc1	Glu-		Araf(1→6)Glu-	ND
	F2	Glu-		Glu-	ND
PPT-	R1	Xyl(1→2)Glu-	Glu-		ND
	Re	Rha(1→2)Glu-	Glu-		ND
	Rg1	Glu-	Glu-		ND
	Rg2	Rha(1→2)Glu-	H-		ND
	Rh1	Glu-	H-		ND

a Final concentration of substrate was 1.0 mM

b The activity against Rd was assumed to be 100%, and corresponded to a specific activity of 10.8 U/mg

c Not detected, specific activity is not detected by the analytical methods used in this study

### Biotransformation of PPD-type ginsenosides by the endoglucanase BcelFp

For the verification of the biotransformation pathways of the four PPD-type ginsenosides (Rb1, Rb2, Rc, and Rd) by using BcelFp, the HPLC analyses were performed. As shown in Fig. 5, ginsenoside Rb1 Rb2, Rc, and Rd had decomposed dramatically after 1 h of reaction. With longer reaction time, still only one product was detected for each substrate (data not shown). Peak 1 and 4 were identified to be GypXVII and F2 by comparison of their retention time with standards, respectively. As lack of standards for peak 2 and 3, their structures were further analyzed by HPLC-MS. As seen in Additional file 1: Fig. S2, The molecular weight of peak 2 was calculated to be 916 u based on its [M-H]^−^ ion at *m/z* 915. The difference between 1078 u (Rb2) and 916 u (peak 2) is 162 u, which corresponds to a glucose moiety. So, BcelFp catalyzed the release of one glucose residue from Rb2 to generate peak 2. As Rb2 has an outer glucose linked to C3 position and an outer arabinopyranose linked to C20 position, the deglycosylation should occur at C3 position of Rb2 to generate CO. Similarly, peak 3 was identified to be CMc1 (See Additional file 1: Fig. S3). Combination with the ^13^ C NMR analysis, product 1–4 were finally confirmed to be GypXVII, CO, CMc1 and F2 (See Additional file 1: Table. S1), respectively. Based on the structural analysis, GypXVII, CO, CMc1, and F2 were generated by hydrolysis of the outer glucose linked to the C3 position of ginsenoside Rb1, Rb2, Rc and Rd, respectively. The biotransformation pathways of ginsenoside Rb1, Rb2 Rc and Rd catalyzed by BcelFp were presented in Fig. 6. In addition, GypXVII, CO, CMc1, and F2 were not further hydrolyzed. Hence, BcelFp didn’t hydrolyze sugar moieties linked to C20 position and the inner glucose residue at C3 position of PPD-type ginsenosides.

As seen in Table 4, the Michaelise-Menten constants (K_m_), Maximum Velocity (V_max_), and catalytic efficiencies (k_cat_/K_m_) for Rb1, Rb2, Rc, and Rd were listed. The order of the k_cat_/K_m_ values of BcelFp for ginsenosides (Rd > Rb1 > Rb2 > Rc) was the same as that observed for relative activity. However, the V_max_ values and the substrate affinity of the enzyme followed the orders Rb1 > Rc > Rb2 > Rd, and Rd > Rb1 > Rb2 > Rc, respectively. BcelFp had higher catalytic efficiency for Rd rather than Rb1, Rb2 and Rc. The k_cat_/K_m_ value of BcelFp for ginsenoside Rd was 27.91 mM^-1^s^-1^, which was higher than that of β-glucosidase from *Gordona terrae* (2.21 mM^-1^s^-1^) (Shin et al. 2015), and β-glycosidase from *Paenibacillus mucilaginosus* (1.78 mM^-1^s^-1^) (Cui et al. 2014).

**Table 4:** Kinetic parameters for BcelFp with PPD-type ginsenosides

Substrate	*K*_*m*_ (µM)	Vmax (mM/h)	*k*_cat_/*K*_*m*_ (s^− 1^ mM^− 1^)
Rb1	3.66 ± 0.04	22.94 ± 2.25	12.53
Rb2	4.02 ± 0.12	18.87 ± 1.56	9.39
Rc	5.95 ± 0.03	22.67 ± 1.79	7.62
Rd	0.67 ± 0.006	9.35 ± 1.37	27.91

## Discussion

The novel endoglucanase gene, termed *BcelFp*, was homologous to GH family 5 in the CAZy classification system. Presently, GH5, one of the largest GH families, exemplifies a family with a large variety of specificities. Until now, 29 known activities of GH5 glycoside hydrolases have been collected at the web of CAZy (Aspeborg et al. 2012). These GH5 enzymes with different substrate specificities share the same retaining catalytic mechanism including one Glu residue as catalytic nucleophile/base and another Glu residue as catalytic proton donor. We can infer from these facts that the diversity in specificity of GH5 proteins may arise by the flexibility in substrate binding sites. The broad specificity of GH5 enzymes makes it possible to further search more different types of substrates and enlarge their application scope. The endoglucanase BcelFp, which exhibited ginsenoside-transforming activity, was a good example of exploring new activities of GH5 glycosidases. All ginsenoside-transforming glycosidases except for arabinofuranosidase and β-galactosidase from *C.saccharolyticus* (Shin et al. 2014) belong to GH 1 or 3 family. BcelFp was the first GH5 enzyme displaying the hydrolysis activity towards ginsenosides. Study of the endoglucanase BcelFp will inspire researchers to identify more and more ginsenoside-transforming glycosidases from GH5 family.

The endoglucanase BcelFp was active and stable under acidic conditions. It exhibited optimal activity at pH 6.0 similar to ginsenoside-transforming glycosidases from thermophilic origins such as β-glucosidases from *Sulfolobus solfataricus* (Noh et al. 2009), *Pyrococcus furiosus* DSMZ 3638 (Oh et al. 2014) and *Thermotoga thermarum* DSM 5069T (Pei et al. 2015). The optimal temperature of BcelFp was about 95 ^o^C, which was higher than the cellulase from *F. nodosum* Rt17-B1(Wang et al. 2010). Even at 95 ^o^C, more than 50% of the enzyme activity remained after incubation for 10 h. The Tm of BcelFp was 96 ^o^C, which was lower than that of β-glycosidase from *Pyrococcus furiosus* (Oh et al. 2014), but higher than that of the other thermophlic glycosidases. Thermostable enzymes serve as ideal catalysts for biotransformation application because high temperatures improve the ginsenosides solubility, enhance the substrate conversion and reduce the need for expensive cooling process. For instance, β-glucosidase from *Thermotoga thermarum* (Pei et al. 2015) transformed ginsenoside Rb1 into Rg3 with a corresponding molar conversion of 97.8% within 60 min at 85 ^o^C. The β-glucosidase from *Thermus thermophilus* (Shin et al. 2014) transformed GypXVII to ginsenoside F2 with a molar yield of 100% at 90 ^o^C.

Biotranformation of ginsenoside Rb1, Rb2, Rc and Rd by glycosidases have been attempted previously. These enzymes can be divided into three groups according to the hydrolysis activity on the linked positions of sugar moieties in ginsenosides. Group I enzymes catalyzed the simultaneous hydrolysis of C3 and C20 sugars in Rb1, Rb2, Rc or Rd. For instance, β-glucosidase from *Actinosynnema mirum* exhibited the hydrolyzing activities as follows: Rb2→CO→CY, Rc→CMc1→CMc and Rd→F2→Rh2→PPD, respectively (Cui et al. 2013). Group II glycosidases catalyze the hydrolysis of C20 sugar moieties in Rb1, Rb2, Rc or Rd. For instance, β-glucosidase from *M.esteraromaticum* hydrolyzed the outer and inner glucoses attached to the C20 position of Rb1 along the following pathway: Rb1→Rd→Rg3 (Quan et al. 2012). Group III enzymes catalyze the hydrolysis of C3 sugars in Rb1, Rb2, Rc or Rd. Table 5 presented a summary concerning the recombinant glucosidases acting on the C3 sugars in Rb1, Rb2, Rc or Rd. Comparing Group I and II, very few Group III enzymes were identified. The endoglucanase BcelFp was proven to be Group III enzyme, as it cleaved the outer glucose moiety at the C3 carbon of ginsenoside Rb1, Rb2, Rc and Rd. The hydrolysis behavior of BcelFp on ginsenosides was similar to the GH1 β-glucosidase from *S. alaskensis* (Shin and Oh 2014) and GH1 β-glucosidase from *C. cellulans* sp. 21 (CcBgl1A) (Yuan et al. 2015). However, BcelFp, CcBgl1A and the *S. alaskensis* glucosidase possessed different physicochemical properties, especially optimal temperature and substrate specificity. BcelFp was thermophilic endoglucanase, while CcBgl1A and the *S. alaskensis* glucosidase were mesophilic β-glucosidase. Moreover, the catalytic efficiency for hydrolysis of ginsenoside Rd by BcelFp (27.91 mM^− 1^ S^− 1^) was higher than that of *S. alaskensis* glucosidase (4.8 mM^− 1^ min^− 1^) and CcBgl1A (8.64 mM^− 1^ S^− 1^) from *C. cellulans* sp. 21.

Besides, a few ginsenoside hydrolyzing recombinant enzymes have been characterized to convert ginsenoside Rd to F2. A β-glucosidase from *Paenibacillus mucilaginosus* KCTC 3870^T^ can hydrolyze Rd into F2 with the catalytic efficiency of 1.78 mM^− 1^ S^− 1^ (Cui et al. 2014). A BglSp from *Sphingomonas* sp. 2F2 showed F2 production abilities from ginsenoside Rd but the low conversion activity limited its application for F2 production (Wang et al. 2011). Moreover, the β-glucosidases from *Penicillium aculeatum* and *Bifidobacterium breve* ATCC 15,700 can hydrolyze Rb1 and Rd into F2, but the enzymes can continuously hydrolyze F2 into C-K (Lee et al. 2013; Zhang et al. 2019). Hence, BcelFp with good thermostability and high catalytic efficiency was a promising biocatalyst for hydrolysis of PPD-type ginsenosides specifically for biotransformation of Rd into F2.

**Table 5:** The recombinant β-glucosidases act on the C3 sugars in Rb1, Rb2, Rc or Rd

Organism	GH	Reaction conditions	Substrate	Product	Cleavage site	Reference
*C. cellulans sp.* 21	1	pH 5.5 35 ℃	Rb1, Rb2, Rc, Rd	GypXVII, CO, CMc1, F2	C-3	Yuan et al. 2015
* S. alaskensis*	1	pH 5.5 50 ℃	Rb1, Rb2, Rc, Rd	Gyp XVII, CO, CMc1, F2	C-3	Shin et al. 2014
*P. aculeatum*	3	pH 4.5 70 ℃	Rb2, Rc, Rd	CO, CY, CMc1, CMc, F2, CK,	C-3	Lee et al. 2013
*B. breve*	3	pH 5.0 35 ℃	Rd	CK, F2	C-3	Zhang et al. 2019

In a conclusion, in the current study, a novel GH5 endoglucanase BcelFp was successfully cloned and expressed in *Escherichia coli*. The recombinant BcelFp exhibited optimal activity at pH 6.0 and 95 ^o^C and showed high thermostability. BcelFp displayed high specificity and catalytic efficiency for biotransformation of ginsenosides Rb1, Rb2, Rc and Rd. Further investigation must be performed on large scale production of GypXVII, CO, CMc1, and F2 by using BcelFp.

## Electronic supplementary material

Below is the link to the electronic supplementary material.

Additional File 1
**Table S1**: ^13^ C NMR dates of the products transformed from Rb1, Rb2, Rc and Rd
**Fig. S1**: Purification of BcelFp. Lane M, molecular mass marker; Lane 1, BcelFp after Ni-NTA affinity chromatography purification
**Fig. S2**: MS/MS spectrum in negative ion mode of product 2 transformed from Rb2 using HPLC-Q-TOF-MS/MS analysis
**Fig. S3**: MS/MS spectrum in negative ion mode of product 3 transformed from Rc using HPLC-Q-TOF-MS/MS analysis

## Data Availability

The datasets supporting the conclusions of this article are included within the article and its Additional file 1.
