# Supplementary material for: Cloning and characterization of thermophilic endoglucanase and its application in the transformation of ginsenosides
Source: AMB Express. 2022 Oct 28;12:136. doi: 10.1186/s13568-022-01473-z (PMC9617004; doi:10.1186/s13568-022-01473-z)

## Supplementary Information

Table S1 ^13^C NMR dates of the products transformed from Rb1, Rb2, Rc and Rd.

| Carbon site | CO | CMc1 | F2 | Gyp XVII |
| --- | --- | --- | --- | --- |
| C-1 | 39.20 | 40.08 | 40.2 | 38.8 |
| C-2 | 26.79 | 27.54 | 27.2 | 25.8 |
| C-3 | 88.82 | 89.72 | 90.6 | 89.3 |
| C-4 | 39.69 | 40.59 | 40.4 | 38.9 |
| C-5 | 56.39 | 57.28 | 57.6 | 56.2 |
| C­-6 | 18.46 | 19.35 | 19.3 | 17.8 |
| C-7 | 35.13 | 36.01 | 35.9 | 34.4 |
| C-8 | 40.06 | 40.95 | 41 | 39.6 |
| C-9 | 50.22 | 51.10 | 51.1 | 49.7 |
| C-10 | 36.97 | 37.86 | 37.9 | 36.5 |
| C-11 | 30.81 | 31.71 | 31 | 30.1 |
| C-12 | 70.18 | 71.14 | 71.9 | 70.1 |
| C-13 | 49.51 | 50.37 | 49.8 | 48.3 |
| C-14 | 51.42 | 52.32 | 52.5 | 51.0 |
| C-15 | 30.72 | 31.61 | 31.6 | 29.4 |
| C-16 | 26.66 | 27.68 | 27.2 | 25.8 |
| C-17 | 51.68 | 52.55 | 53.1 | 51.5 |
| C-18 | 16.29 | 17.19 | 16.3 | 14.9 |
| C-19 | 16.03 | 16.91 | 16.8 | 15.3 |
| C-20 | 83.50 |  | 84.9 | 83.6 |
| C-21 | 22.35 | 23.28 | 22.9 | 21.1 |
| C-22 | 36.20 | 37.07 | 36.7 | 35.4 |
| C-23 | 23.23 | 24.08 | 24.3 | 22.5 |
| C-24 | 125.97 | 126.95 | 125.9 | 124.6 |
| C-25 | 131.12 | 131.93 | 132.3 | 130.8 |
| C-26 | 25.81 | 26.70 | 25.9 | 24.6 |
| C-27 | 17.90 | 18.78 | 18 | 16.6 |
| C-28 | 28.16 | 29.06 | 28.4 | 27.0 |
| C-29 | 16.82 | 17.71 | 16.8 | 15.4 |
| C-30 | 17.42 | 18.29 | 17.2 | 16.0 |
| 1. Glucopyranosyl (inner) |  |  |  |  |
| 1 | 107.00 | 107.90 | 106.8 | 105.3 |
| 2 | 75.82 | 75.96 | 75.4 | 73.9 |
| 3 | 78.41 | 80.20 | 78.2 | 76.9 |
| 4 | 71.94 | 73.04 | 71.2 | 70.3 |
| 5 | 78.80 | 79.32 | 77.7 | 76.3 |
| 6 | 63.14 | 64.03 | 62.5 | 61.3 |
| 20-Glucopyranosyl (inner) |  |  |  |  |
| 1 | 98.16 | 99.03 | 98.3 | 96.7 |
| 2 | 74.93 | 75.96 | 75.6 | 73.7 |
| 3 | 79.27 | 79.75 | 78.3 | 77.1 |
| 4 | 71.85 | 72.83 | 71.6 | 70.2 |
| 5 | 76.79 | 77.50 | 77.9 | 74.2 |
| 6 | 69.25 | 69.43 | 62.8 | 68.8 |
| 20-Glucopyranosyl(outer) |  |  |  |  |
| 1 |  |  |  | 103.6 |
| 2 |  |  |  | 76.5 |
| 3 |  |  |  | 75.4 |
| 4 |  |  |  | 70.2 |
| 5 |  |  |  | 76.5 |
| 6 |  |  |  | 61.3 |
| 20-Arabinofuranosyl |  |  |  |  |
| 1 | 104.69 | 111.06 |  |  |
| 2 | 72.18 | 84.30 |  |  |
| 3 | 74.17 | 79.70 |  |  |
| 4 | 68.62 | 86.92 |  |  |
| 5 | 65.63 | 63.56 |  |  |
| 6 |  |  |  |  |

**Fig. S1** Purification of BcelFp. Lane M, molecular mass marker; Lane 1, BcelFp after Ni-NTA affinity chromatography purification;


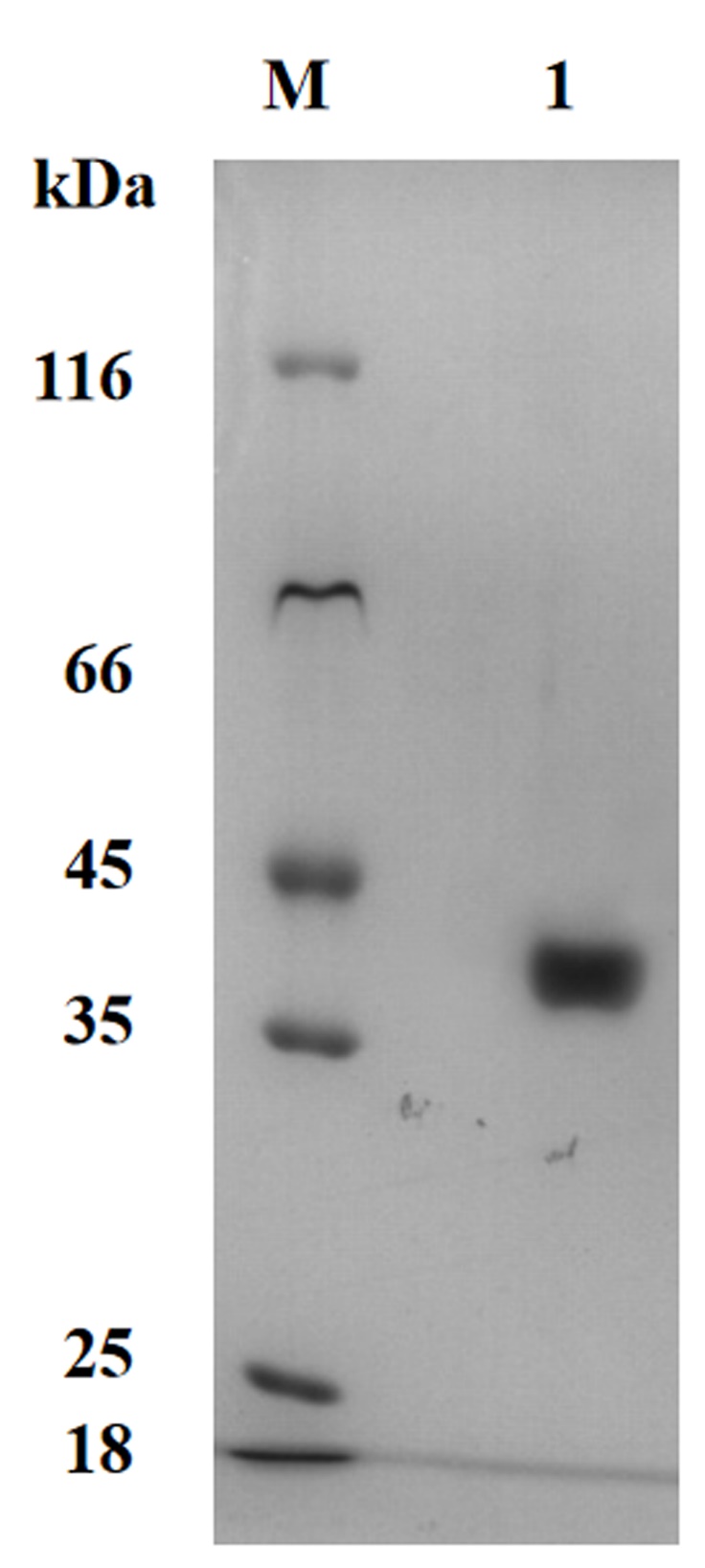


**Fig. S2** MS/MS spectrum in negative ion mode of product 2 transformed from Rb2 using HPLC-Q-TOF-MS/MS analysis;


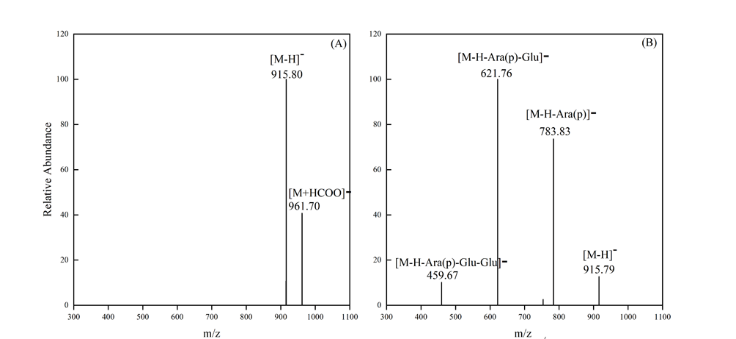


**Fig. S3**; MS/MS spectrum in negative ion mode of product 3 transformed from Rc using HPLC-Q-TOF-MS/MS analysis;


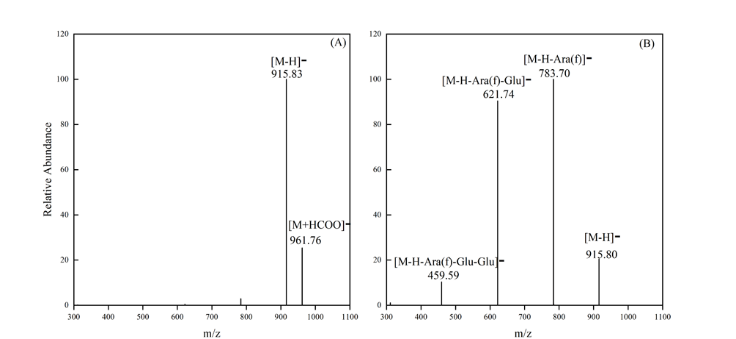

Supplement: Supplementary file 1 — Additional File 1 Table S1: 13 C NMR dates of the products transformed from Rb1, Rb2, Rc and Rd Fig. S1: Purification of BcelFp. Lane M, molecular mass marker; Lane 1, BcelFp after Ni-NTA affinity chromatography purification Fig. S2: MS/MS spectrum in negative ion mode of product 2 transformed from Rb2 using HPLC-Q-TOF-MS/MS analysis Fig. S3: MS/MS spectrum in negative ion mode of product 3 transformed from Rc using HPLC-Q-TOF-MS/MS analysis [file 13568_2022_1473_MOESM1_ESM.docx]
